# Cuticular Hydrocarbon Profiles of Himalayan Bumble Bees (Hymenoptera: *Bombus* Latreille) are Species-Specific and Show Elevational Variation

**DOI:** 10.1007/s10886-024-01486-x

**Published:** 2024-03-12

**Authors:** Jaya Narah, Martin Streinzer, Jharna Chakravorty, Karsing Megu, Johannes Spaethe, Axel Brockmann, Thomas Schmitt

**Affiliations:** 1 Rajiv Gandhi University, Papum Pare, Arunachal Pradesh India; 2 National Centre for Biological Sciences - Tata Institute of Fundamental Research, Bengaluru, Karnataka India; 3 University of Vienna, Vienna, Austria; 4 Dera Natung Government College, Itanagar, Arunachal Pradesh India; 5 Department of Behavioral Physiology and Sociobiology, Biocentre, University of Würzburg, Würzburg, Germany; 6 Department of Animal Ecology and Tropical Biology, Biocentre, University of Würzburg, Würzburg, Germany

**Keywords:** CHC Profiles, Desiccation Stress, Adaptation, Climatic Conditions

## Abstract

Bumble bees are important pollinators in natural environments and agricultural farmlands, and they are in particular adapted to harsh environments like high mountain habitats. In these environments, animals are exposed to low temperature and face the risk of desiccation. The Eastern Himalayas are one of the recognized biodiversity hotspots worldwide. The area covers subtropical rainforest with warm temperature and high precipitation as well as high mountain ranges with peaks reaching up to 7,000 m, shaping a diverse floral and faunal community at the different elevational zones. To identify possible adaptation strategies, we investigated the cuticular hydrocarbon profiles of four bumble bee species occurring at different elevational ranges in Arunachal Pradesh, the northeastern most state in India. At 17 locations along an elevational gradient, we collected workers of two species from lower elevations (*B. albopleuralis* and *B. breviceps;* ~ 100 m − 3,000 m asl) and two species from higher elevations (*B. prshewalskyi* and *B. mirus*; ~ 2,800 m − 4,500 m asl). The CHC profiles of all four species showed a significant degree of variation in the composition of hydrocarbons, indicating species specificity. We also found clear correlation with elevation. The weighted mean chain length of the hydrocarbons significantly differed between the low and high elevation species, and the proportion of saturated hydrocarbons in CHC profiles significantly increased with the elevational range of the bumble bee species. Our results indicate that bumble bees living at high elevations reduce the risk of water loss by adapting their CHC composition on their cuticle, a phenomenon that has also been found in other insects like ants and fruit flies.

**Supplementary Information:**

The online version contains supplementary material available at 10.1007/s10886-024-01486-x.

## Introduction

Bumble bees, which are adapted to moderate and cold temperate environments, occur in India only in the mountain ranges of the Himalayas where they play an important role as pollinators of plants and agricultural crops (Rather et al. 2023). The Himalayas are one of the longest mountain ranges in the world and exhibit a steep climatic gradient from the colder and dry western part to the warmer and very humid eastern region. Corresponding to this gradient, one finds a strong shift in floral composition along the mountain range, from temperate broadleaf forest and arid alpine meadows in the west to wet subtropical broadleaf forest and moist alpine meadows in its east (Rawat 2017). In the eastern part, precipitation can reach up to 5,000 mm per year, but decreases with elevation (Dhar and Nandargi 2006). Temperature and rainfall are environmental variables that significantly affect the presence and distribution of species. Due to global climate change, these local ecosystems might face dramatic transformations in the next decades that in turn affect local flora and fauna (Shrestha et al. 2012). For the conservation of Himalayan ecosystems, we need to know the distribution and life histories of plants and animals, and their potential plasticity to respond and adapt to climate changes (Kerr et al. 2015). Due to their importance as pollinators of many flowering plants, bumble bees and other wild bees became model systems to explore their vulnerability and resilience against climate changes (Soroye et al. 2020; Maebe et al. 2021; Warrit et al. 2023). Possible adaptations include, for example, changes in pilosity (Hines et al. 2022), cuticular hydrocarbon composition (Maihoff et al. 2023), temperature tolerance (Pimsler et al. 2020; Martinet et al. 2021), respiratory and neural systems (Jackson et al. 2020).

The mountain ranges of the Eastern Himalayas in Arunachal Pradesh (India) exhibit one of the largest elevational spans worldwide, from 44 m asl close to the Brahmaputra riverbed up to 7,060 m asl of the highest peak. This geographical peculiarity provides a unique opportunity to study bumble bee traits that are related to different elevation and climatic factors, like temperature and humidity, by comparing morphological and physiological characters of species or populations with restricted elevation ranges. Beside the fact that this part of the Himalayas is one of the most biodiverse regions on our planet (Myers et al. 2000), it is also one of the most understudied areas. Thus, we recently started a systematic survey of the bumble bee fauna in this region (Streinzer et al. 2019).

In the present study we compared the cuticular hydrocarbon (CHC) profiles of two pairs of bumble bee species, which only occur in the lower (*B. albopleuralis*, *B. breviceps*) or higher elevations (*B. prshewalskyi*, *B. mirus*) (Streinzer et al. 2019). It is known that CHC profiles of insects are a highly variable phenotypic character. As the boundary between the organism and the environment they play a vital role in protecting the body against detrimental abiotic environmental conditions, like heat and desiccation stress, but they also function as cues and signals in intra- and interspecific recognition and communication (Blomquist et al. 2021; Maihoff et al. 2023; Sprenger and Menzel 2020). The CHC profiles can be composed of more than 100 compounds from three substance classes, n-alkanes, methyl-branched alkanes, and unsaturated hydrocarbons (olefins), with different physico-chemical properties. Due to these differences, the profiles can adapt to extreme environmental conditions, such as drought stress, by alternating the relative composition of compounds with different structural features and chain-lengths (Menzel et al. 2018). For example, reducing the relative number of unsaturated hydrocarbons in favor of n-alkanes will harden the CHC profile and make the hydrophobic wax layer of the cuticle less water permeable. A similar effect can be achieved by an increase of chain-length of the CHC compounds (Gibbs and Rajpurohit 2010; Menzel et al. 2017). To investigate whether bumble bees living in the high elevational habitats of the Eastern Himalayas have adapted their CHC profile to the local conditions, we compared CHC composition of high elevation and low elevation species. We hypothesized that species from high elevations possess CHCs with a higher proportion of saturated hydrocarbons and show an increase in the medium chain-length compared to species from lower elevations because more drought stress is expected due to lower humidity in higher elevations than in lower elevations of the eastern range of the Himalaya (Dhar and Nandargi 2006).

## Materials and methods

### Study Area and Bumble bee Collection

**Fig. 1:**
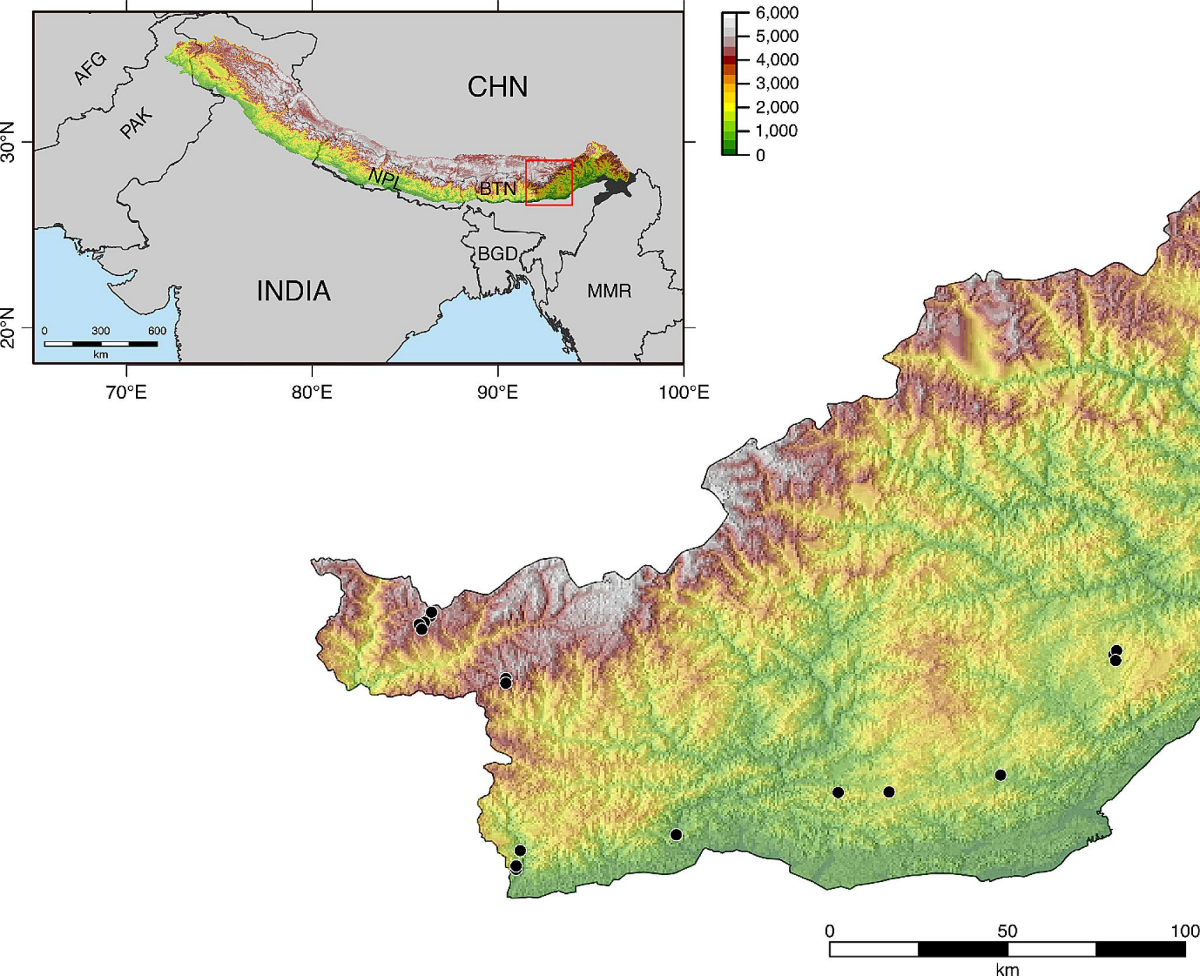
Sampling locations in Arunachal Pradesh.Overview map of the region with an overlay of the elevation profile of the Himalayan Mountain range. The red box indicates the enlarged part of Arunachal Pradesh shown at the bottom. Sampling locations (*N* = 17) are shown as black dots. Color refers to elevation above sea level. Elevation data from Jarvis et al. (2008), country borders from Runfola et al. (2020) and Himalaya range borders from Liu and Zhu (2022). (For detailed information about the location and number of collected specimens see Suppl. Table 1)

Foraging bumble bee workers were caught at 17 locations at different elevations, ranging from 150 m to 4,403 m asl, in the western districts of Arunachal Pradesh between June and September 2021 (Fig. 1). We investigated the CHC profiles of two pairs of low (wet subtropical broadleaf forest) and high elevation (moist alpine meadows) species: *Bombus albopleuralis* Friese, 1916 (range: 150–1,561 m, *N* = 31), *Bombus breviceps* Smith, 1852 (range: 941–1,575 m, *N* = 12), *B. mirus* (Tkalcu, 1968) (range: 4,136–4,403 m, *N* = 29), and *B. prshewalskyi* Morawitz, 1880 (range 4,112–4,403 m, *N* = 32) (see supplementary Table 1). Collected specimens were brought to the lab and stored at -20 °C until CHCs were extracted.

### Chemical Analyses

CHC profiles were extracted from each worker by immersing the specimen in n-hexane for 10 min after removing the pollen from the hind legs of the bees. The CHC extracts were stored at -20 °C and later reconstituted in approximately 150 µl of hexane for the gas chromatography–mass spectrometry (GC-MS) analysis. The extracts were analyzed with an Agilent 7890 gas chromatography coupled with an Agilent 5975 Mass Selective Detector (GC-MS, Agilent, Waldbronn, Germany). The GC (split/splitless injector in splitless mode for 1 min, injected volume 1 µl at 300 °C) was equipped with a DB-5 fused silica capillary column (30 m × 0.25 mm ID, df = 0.25 μm; J&W Scientific, Folsom, United States). Helium served as carrier gas with a constant flow of 1 mL/min. The following temperature program was used: start temperature 60 °C, temperature increase by 5 °C per min up to 300 °C, isotherm at 300 °C for 10 min. The electron ionization mass spectra (EI-MS) were acquired at an ionization voltage of 70 eV (source temperature: 230 °C). Chromatograms and mass spectra were recorded with the software HP Enhanced ChemStation G1701AA (version A.03.00; Hewlett Packard, Palo Alto, United States). Alongside CHC samples, we ran two analytical alkane standards (C_8_-C_20_ and C_21_-C_40_; Sigma Aldrich, St. Louis, United States) for the calculation of the retention indices. CHC compounds were quantified by integrating peak areas and calculating their relative composition. The identification was based on the compound specific retention index and the diagnostic fragmentation pattern (Carlson et., al. 1998). DMDS derivatization was used to identify the double-bond positions in unsaturated hydrocarbons if possible (Carlson et al. 1989).

### Statistical Analysis

All statistical analyses were performed using R software (version 4.3.0; R core team 2023). We compared the relative abundance of compounds in the CHC profiles of the workers among all four bumble bee species. CHC compounds were assessed by non-metric multidimensional scaling (NMDS), a two-dimensional ordination method to visualize similarities. Bray-Curtis distance method was used to calculate the dissimilarities between the workers’ profiles. CHC profile composition differences between species were calculated using permutational multivariate analysis of variance (PERMANOVA) in the package vegan (Oksanen et al. 2020) and PairwiseAdonis (Martinez Arbizu 2020) with Bonferroni correction. Permutations were set on 9,999. We calculated the weighted mean chain length of a CHC profile based on the abundance of each hydrocarbon. Differences in weighted mean chain length and proportion of saturated CHCs were analyzed with Kruskal Wallis test and multiple comparisons were performed with Dunn’s test in R (Dunn 1964; Dinno 2015).

### COI Barcoding and Species Identification

As species identification based on morphological traits is difficult for bumble bees of the Eastern Himalayas, we used DNA barcoding as an additional method for identification (Hebert et al. 2003). DNA was extracted from the muscles of the forelegs or middle legs and isolated using DNeasy Blood & Tissue extraction kit (Qiagen, Venlo, The Netherlands) following the manufacturer’s instructions with slight modifications. The amplification of the standard barcode COI region by means of polymerase chain reaction (PCR) was performed using standard primers (LepF1: 5´-TTCAACCAATCATAAAGATATTGG-3´ and LepR1: 5´-AACTTCTGGATGTCCAAAAAATCA-3´; Hebert et al. 2004). PCR reactions had a volume of 25 µl and contained 0.167 U/µl Taq DNA Polymerase (Invitrogen, Walthem, United States), 1X Taq Buffer (Invitrogen), 1 mM MgCl_2_ (Invitrogen), 0.25 mM dNTP mix (Invitrogen), 0.4 µM LepF1, 0.4 µM LepR1, 16.29 µl nuclease free water and 3 µl template DNA. PCR products were verified by gel electrophoresis using 3 µl of PCR product and 2 µl of 100 bp Plus DNA Ladder (Biolabs, Ipswich, Unites States) and run at 90 V for 1 h. The gel was then inspected under UV illumination in a Gel-Doc. The purified PCR products were submitted to Next Generation Genomics Facility at Bangalore LifeScience Cluster and sequenced in both directions. The complete COI barcode sequence was assembled using sequences of both strands by means of BioEdit (version 7.2 for Windows) or AliView (version 1.2) using MUSCLE algorithm (Edgar 2004). The final alignment was 657 bp long after the primer sequences were removed.

Reference sequences of target species and closely related taxa were selected from Santos Júnior et al. (2022) and downloaded from the BOLD database (Ratnasingham and Hebert 2007). If available, sequences from revision studies and designated barcode proxy type specimens were given preference (Williams et al. 2020, 2023). For species with no published reference sequences, we used sequences from specimens stored at the NCBS research collection facility, which were identified based on morphology (Streinzer et al. 2019). A phylogenetic tree was reconstructed using the Maximum Likelihood method using the GTR + GI model (Nei and Kumar 2000; Kumar et al. 2018) for all three codon positions (Kimura 1980) in MEGA (version 10.2 for Windows). Confidence values for nodes were calculated using the bootstrap method with 1,000 replicates (Felsenstein 1985). FigTree V1.4.4 was used to view and edit the phylogenetic tree.

## Results

### Species Identification

All specimens could be morphologically identified to species level and were sequenced for confirmation of their identity. We obtained sequences for all *B. albopleuralis* (*N* = 31) and *B. breviceps* (*N* = 12), c. 75% of *B. mirus* (*N* = 22, out of 29), and c. 60% of *B. prshewalskyi* (*N* = 19 out of 32). Comparison of the barcode sequences with published sequences and reference sequences from specimens in the NCBS collection confirmed the morphological identification of *Bombus albopleuralis, B. mirus and B. prshewalskyi.* For *B. breviceps*, all sequences were likely low-divergence non-orthologous barcodes (numts). Numts are a known issue with the subgenus *Alpigenobombus* (Williams et al. 2023). Based on the position of the sequences in the phylogenetic tree and the morphological identification, the species identity is not in doubt.

### CHC Profiles

**Fig. 2:**
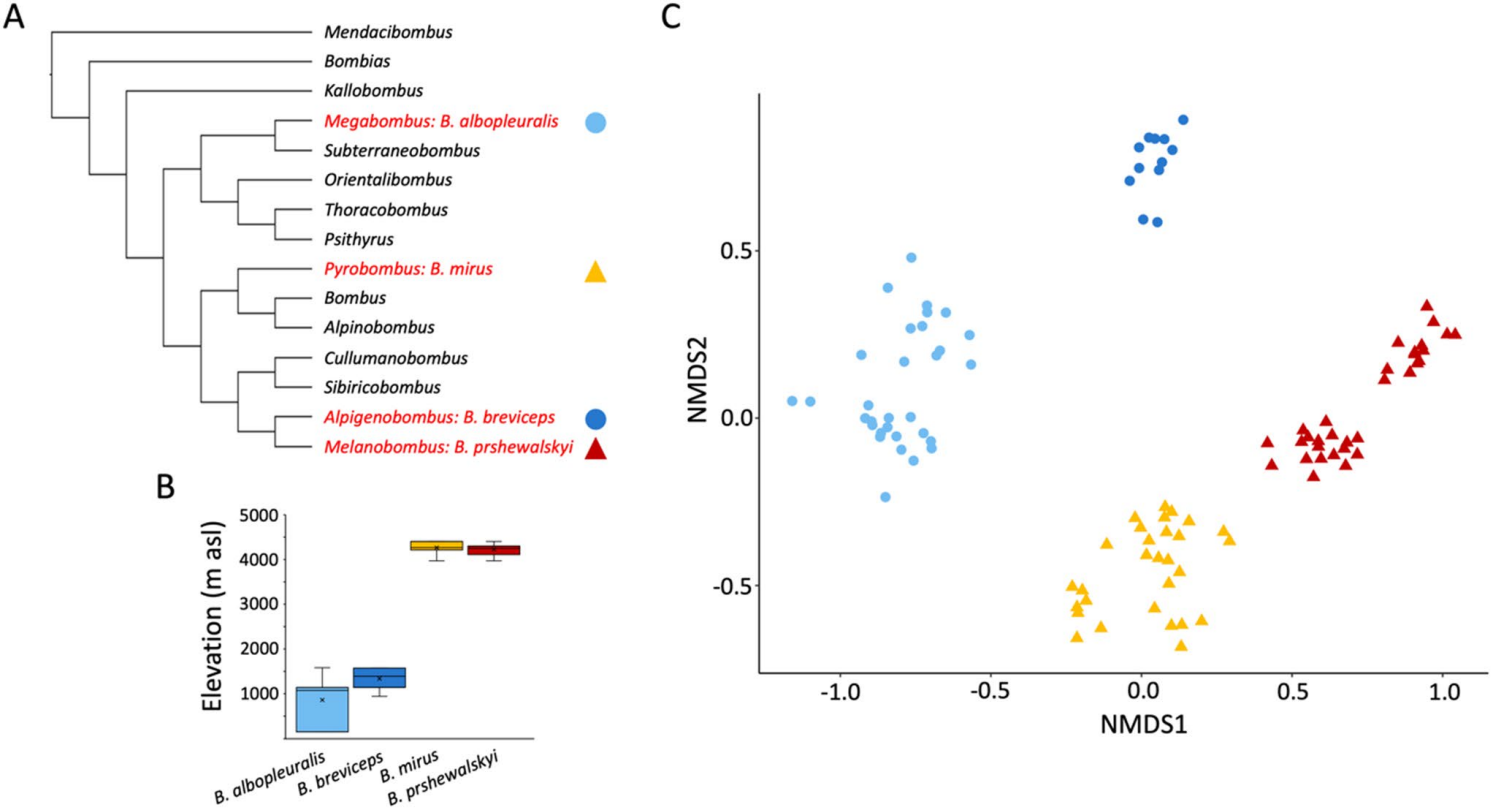
CHC profile diversity of four Himalayan bumble bee species with different elevational ranges. (**A**). Phylogenetic position of the study species embedded in the subgeneric classification of the bumble bees (Sun et al. 2021, Williams et al., 2022). (**B**) Elevational range of collected bumble bee specimens (box indicating first and third quartile and median and whiskers the total range). (**C**) Diversity of CHC profiles of bumble bees displayed in a two-dimensional graph by non-metric multidimensional scaling (NMDS) based on Bray-Curtis distances. Distance between symbols indicates the degree of similarity among the CHC profiles. Each symbol represents the CHC profile of an individual bumble bee worker. Circles: species with lower elevational ranges, triangles: species with higher elevational ranges. Colors indicate the species

The composition of CHC profiles consisting of n-alkanes, monomethyl-branched alkanes, alkenes, and alkadienes significantly differed among the four species (PERMANOVA: F = 143.39, df = 3, *p* < 0.001, R^2^ = 0.809), resulting in species specific CHC profiles (Fig. 2; Suppl. Figure 1; Suppl. Tables 2, 3; PERMANOVA between all species pairs: *p* < 0.001).

**Fig. 3:**
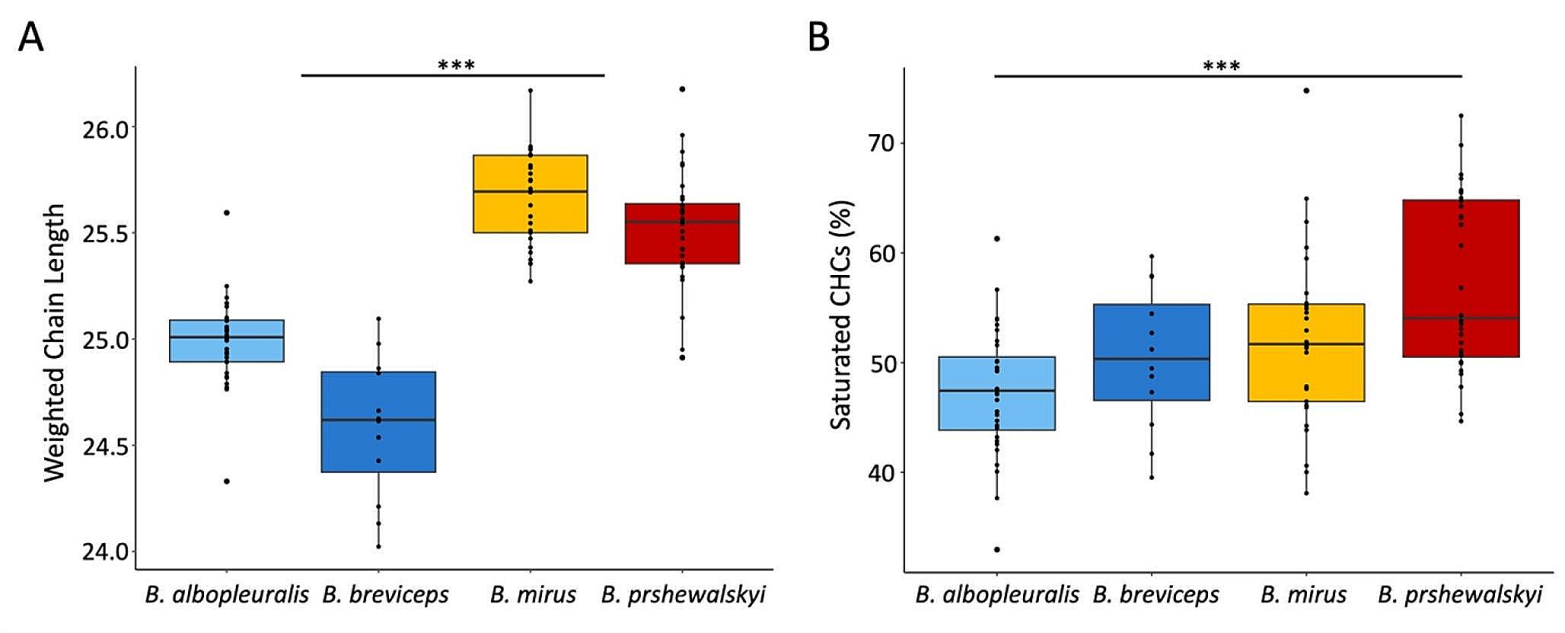
Differences in elevation associated chemical traits of CHC profiles in the four bumble bee species. Differences in (**A**) weighted mean chain lengths of the hydrocarbons and (**B**) the proportion of saturated hydrocarbons in the CHC profiles. Significance values refer to the results of Dunn’s test for multiple comparisons (***, *p* < 0.001; see main text and Suppl. Tables 4 & 5)

The weighted mean chain lengths of hydrocarbons in the CHC profiles significantly differed between species (H = 72.165, df = 3, *p* < 0.001). Post-hoc comparisons showed that each individual species differed significantly from both species occurring in the other elevation group (*p* < 0.001 in all cases, Suppl. Table 4), but not from the other in the same group (*p* > 0.025; P-levels adjusted for multiple comparisons; Fig. 3a, Suppl. Table 4).

The CHC profiles of lower elevation species consisted of hydrocarbons with shorter mean chain length (*B. albopleuralis*, mean: 24.9 ± 0.2 and *B. breviceps*, mean: 24.5 ± 0.33) whereas the CHC profiles of the higher elevation species contained hydrocarbons with significantly higher weighted mean chain length (*B. mirus*, mean: 25.6 ± 0.21 and *B. prshewalskyi*, mean: 25.5 ± 0.26).

The proportion of saturated CHC components in the CHC profile differed significantly among species (H = 22.521, df = 3, *p* < 0.001). Post-hoc comparison between all species pairs showed that only *B. albopleuralis* and *B. prshewalskyi* differed significantly in the amount of saturated CHC components (Fig. 3b, Suppl. Table 5).

The proportion of saturated CHC components showed a trend to increase with the elevational range of the bumble bee species (Fig. 3b). The species with the lowest elevational range, *B. albopleuralis*, had the lowest mean proportion of saturated CHC components (average: 47 ± 5.7%) followed by *B. breviceps* (average: 50 ± 6.4%), and the two high elevation species, *B. mirus* (average: 52 ± 7.8%) and *B. prshewalskyi* (average: 57 ± 7.9%).

## Discussion

In this study, we investigated the variation in CHC profiles of four bumble bee species with different elevational distributions to check for possible adaptations to habitat-specific climatic conditions. Bumble bees often occur across a wide elevational range, but only a few species occur in (sub-)tropical lowlands, where the conditions are typically not suitable for these bees that are mostly adapted to cold environments (Moure and Sakagami 1962; Williams 1991, 2022; Gonzalez et al. 2004). In the mountain regions of Arunachal Pradesh, the highest bumble bee diversity occurs in the subalpine zone between 3,000 and 4,000 m asl and significantly decreases towards lower elevations (Streinzer et al. 2019). We found that species preferring high or low elevational niches possess distinct and species-specific CHC profiles. These differences are particularly pronounced by the presence of methyl-branched alkanes in the two high elevation species, whereas these compounds are absent in the two low elevation species (Suppl. Table 2). In addition to the species-specificity of CHC profiles, the ordination of the data also suggests subclusters in all species except *B. breviceps* (see Fig. 2). Since these subclusters are composed of specimens collected at different locations, they may present different populations (although this was not statistically tested). Most of our collection sites followed river valleys or mountain passes since these are the only possibilities to reach remote areas. These valleys are steep and may act as distribution barriers, and thus the CHC subclusters might be the result of genetic drift due to reduced or absent gene flow between these distinct populations. However, a more extensive collection of specimens and a population genetic study is needed to understand the gene flow among populations and the related CHC profiles in more detail.

The compositional features of the CHC profiles are correlated with similarities in the elevational niche preferences of the corresponding species, suggesting that CHCs found on the cuticle of these species might have been shaped by environmental factors correlating with elevation (e.g., temperature, precipitation, humidity) rather than phylogeny, since all investigated species are from different subgenera (Fig. 2A; Williams 2022). High elevation habitats are often drier, with less precipitation and less humidity than habitats at lower elevations. This is particularly true for the eastern range of the Himalayas (Dhar and Nandargi 2006). To cope with drought stress, insects are in principle able to harden their CHC by developmentally changing the composition (Stinziano et al. 2015; Rajpurohit et al. 2017) or evolving a genetically fixed adaptation (Menzel et al. 2017; Sprenger and Menzel 2020). Mechanistically, CHC profiles can be hardened either by elongating the chain-length of their compounds or by changing the relative composition towards a higher proportion of saturated versus unsaturated hydrocarbons. In our study, we found both traits realized. The chain-length is elongated in the high elevation species compared to both low elevation species. The variation of the ratio of saturated to unsaturated hydrocarbons is less pronounced among all investigated species, and significantly increased only when comparing *B. albopleuralis* as a low elevation species with B. *prshewalskyi* as a high elevation species (Fig. 3).

The results of our study are congruent with a recent study of halictid bees on the slope of Mount Kilimanjaro. Bees of the genus *Lasioglossum* showed a change in the composition of their CHC profiles similar to our bumble bees along an elevational gradient between 830 m and 3,780 m asl (Mayr et al. 2021). In contrast, a study on bumble bees from an elevational gradient in the European Alps did not find any correlation of the CHC profiles with potential drought stress at higher elevations (Maihoff et al. 2023). However, in this study the elevational gradient was only 1,000 m and did not reach the alpine level, where climatic conditions are expected to be most extreme. Although we did not test the actual desiccation stress for the high elevation species in our study, we hypothesize that the species-specific differences in CHC profiles of the Himalayan bumble bee species are an adaptation to the local climatic conditions. Alternatively, the species-specific CHC profiles could be the result of genetic drift. Similar results have been shown by comparing large numbers of ant species from the genus *Camponotus* and *Drosophila* species from habitats with different climatic conditions (Menzel et al. 2017; Wang et al. 2022). However, with our approach we cannot finally discriminate between an effect of genetically fixed adaptation and of a plastic reaction to the environmental conditions. Several studies in *Drosophila*, for example, demonstrated that the hardening of a CHC profile is a direct reaction to desiccation stress (Stinziano et al. 2015; Rajpurohit et al. 2017).

To summarize, our results provide evidence that differences in the CHC profiles of bumble bee species from the Northeastern slope of the Himalayas are correlated with climatic differences of high and low elevational habitats. Future studies should include the CHC analysis of more species from low and high elevations and in particular more elevational generalist. For example, *B. haemorrhoidalis*, a common species in this area, covers a huge elevational range from 400 m to almost 3,500 m asl (Streinzer et al. 2019), and thus seems to be able to cope with various climatic conditions.

## Electronic Supplementary Material

Below is the link to the electronic supplementary material.

Supplementary Material 1

Supplementary Material 2

## Data Availability

No datasets were generated or analysed during the current study.
