# Supplementary material for: Cuticular Hydrocarbon Profiles of Himalayan Bumble Bees (Hymenoptera: Bombus Latreille) are Species-Specific and Show Elevational Variation
Source: J Chem Ecol. 2024 Mar 12;50(12):969–77. doi: 10.1007/s10886-024-01486-x (PMC11717848; doi:10.1007/s10886-024-01486-x)
Supplement: Supplementary file 1 — Supplementary Material 1 [file 10886_2024_1486_MOESM1_ESM.docx]

**Supplementary figure 1a:** Chromatogram of the CHC profile of *Bombus albopleuralis*. Peak numbers correspond to peak numbers in supplementary table 2.

**44**

**46**

**30**

**28**

**24**

**13**

**23**

**8**

**15**

**Supplementary figure 1b:** Chromatogram of the CHC profile of *Bombus breviceps*. Peak numbers correspond to peak numbers in supplementary table 2.

**65**

**58**

**46**

**30**

**28**

**15**

**13**

**2**

**4**

**Supplementary figure 1c:** Chromatogram of the CHC profile of *Bombus mirus*. Peak numbers correspond to peak numbers in supplementary table 2.

**58**

**55**

**46**

**43**

**30**

**27**

**15**

**12**

**4**

**Supplementary figure 1d:** Chromatogram of the CHC profile of *Bombus prshewalskyi*. Peak numbers correspond to peak numbers in supplementary table 2.

**65**

**58**

**55**

**46**

**43**

**30**

**27**

**15**

**12**
