# Supplementary material for: Cuticular Hydrocarbon Profiles of Himalayan Bumble Bees (Hymenoptera: Bombus Latreille) are Species-Specific and Show Elevational Variation
Source: J Chem Ecol. 2024 Mar 12;50(12):969–77. doi: 10.1007/s10886-024-01486-x (PMC11717848; doi:10.1007/s10886-024-01486-x)
Supplement: Supplementary file 2 — Supplementary Material 2 [file 10886_2024_1486_MOESM2_ESM.docx]

**SUPPLEMENTARY MATERIAL**

**Supplementary Table 1**: List of collected worker specimens.

| **Elevation (m)** | **Species** | **Number** | **Locality** |
| --- | --- | --- | --- |
| 150 | *B. albopleuralis* | 13 | N27°4.215', E092°35.472'  Bhalukpong, West Kameng |
| 800 | *B. albopleuralis* | 10 | N26°58.290', E092°7.972'  Balemu, West Kameng |
| 941 | *B. breviceps* | 2 | N27°14.465', E093°31.144'  Toru, Sagalee, Papum Pare |
| 1145 | *B. albopleuralis* | 5 | N27°11.462', E093°3.261'  Papu Valley, West Kameng |
|  | *B. breviceps* | 2 |  |
| 1217 | *B. breviceps* | 2 | N27°1.482', E092°8.674'  Ankalin, West Kameng |
| 1445 | *B. albopleuralis* | 1 | N27°11.542', E093°11.997'  10 km ahead Pakke Kessang, |
| 1561 | *B. albopleuralis* | 2 | N27°35.126’, E093°50.598’  Kalung Village, Ziro Valley |
|  | *B. breviceps* | 2 |  |
| 1575 | *B. breviceps* | 4 | N27°35.817’, E093°51.47'  Salaya, Ziro Valley |
|  |  |  |  |
| 4112 | *B. prshewalskyi* | 12 | N27°31.011', E092°6.203'  Sela Pass, West Kameng |
| 4136 | *B. mirus* | 5 | N27°39.478', E091°51.778'  Nagula, Tawang |
| 4215 | *B. prshewalskyi* | 2 | N27°30.228', E092°6.205'  Sela top, West Kameng |
| 4247 | *B. prshewalskyi* | 7 | N27°40.666', E091°52.202'  Klemta Check gate, Tawang |
|  | *B. mirus* | 9 |  |
| 4264 | *B. prshewalskyi* | 2 | N27°40.299', E091°51.339'  10km N Tawang, Tawang |
|  | *B. mirus* | 7 |  |
| 4300 | *B. prshewalskyi* | 10 | N27°41.745', E91°53.260'  Klemta, Tawang |
|  | *B. mirus* | 1 |  |
| 4403 | *B. mirus* | 7 | N27°42.412', E091°53.455'  Bumla Pass, Tawang |

**Supplementary Table 2**. List of peaks of cuticular hydrocarbons from low and high elevation bumble bee species. All n-alkanes were verified using commercially available standards.

| **#** | **Compound** | **RI** | ***B. albopleuralis*** | ***B. breviceps*** | ***B. mirus*** | ***B. prshewalskyi*** |
| --- | --- | --- | --- | --- | --- | --- |
| 1 | 9-C21ene | 2074 | - | 0.430 ±0.047 | 0.024 ±0.008 | 0.182 ±0.024 |
| 2 | 7-C21ene | 2081 | 0.578 ±0.101 | 4.972 ±0.317 | - | 0.072 ±0.015 |
| 3 | 5-C21ene | 2088 | - | 1.067 ±0.099 | - | - |
| 4 | C21 |  | 0.343 ±0.064 | 3.538 ±0.198 | 1.098 ±0.066 | 0.837 ±0.059 |
| 5 | 9-C22ene | 2175 | - | - | 0.016 ±0.007 | 0.123 ±0.013 |
| 6 | 7-C22ene | 2182 | 1.069 ±0.195 | 0.981 ±0.078 | - | - |
| 7 | 5-C22ene | 2188 | - | 0.106 ±0.019 | - | - |
| 8 | C22 |  | 1.321 ±0.295 | 0.935 ±0.164 | 0.132 ±0.011 | 2.060 ±0.395 |
| 9 | 2-MeC22 | 2263 | - | - | 0.002 ±0.002 | - |
| 10 | 7,15-C23diene | 2258 | 0.014 ±0.005 | - | - | - |
| 11 | 5,15-C23diene | 2268 | 0.331 ±0.051 | - | - | - |
| 12 | 9-C23ene | 2270 | 0.193 ±0.044 | 7.915 ±0.538 | 7.405 ±0.340 | 12.002 ±0.579 |
| 13 | 7-C23ene | 2277 | 2.272 ±0.157 | 16.488±0.849 | 0.381 ±0.036 | 0.576 ±0.048 |
| 14 | 5-C23ene | 2287 | 0.250 ±0.022 | 2.098 ±0.207 | - | - |
| 15 | C23 |  | 16.701 ±0.784 | 16.207±0.556 | 10.005 ±0.379 | 12.369 ±0.287 |
| 16 | 11-; 9-MeC23 | 2335 | - | - | 0.015 ±0.006 | 0.430 ±0.043 |
| 17 | 7-MeC23 | 2342 | - | - | 0.007 ±0.004 | 0.177 ±0.025 |
| 18 | 5-MeC23 | 2351 | - | - | - | 0.224 ±0.035 |
| 19 | C24diene | 2365 | 0.031±0.009 | - | - | - |
| 20 | 9-C24ene | 2372 | - | 0.290 ±0.055 | 0.390 ±0.021 | 0.431 ±0.072 |
| 21 | 7-C24ene | 2380 | 0.743 ±0.038 | 0.347 ±0.027 | - | - |
| 22 | 5-C24ene | 2390 | 0.489 ±0.080 | 0.344 ±0.086 | - | - |
| 23 | C24 |  | 1.741 ±0.144 | 0.824 ±0.092 | 0.312 ±0.018 | 1.502 ±0.225 |
| 24 | 7,17-C25diene | 2458 | 6.941 ±0.832 | - | 0.110 ±0.020 | - |
| 25 | 5,17C25diene | 2467 | 0.356 ±0.096 | - | - | - |
| 26 | 11-C29ene | 2468 | - | - | 2.353 ±0.923 | - |
| 27 | 9-C25ene | 2471 | 1.730 ±0.636 | 2.677 ±0.193 | 18.355 ±1.141 | 16.883 ±0.535 |
| 28 | 7-C25ene | 2482 | 20.243 ±1.064 | 4.992 ±0.199 | 1.078 ±0.164 | 0.775 ±0.092 |
| 29 | 5-C25ene | 2490 | 3.023 ±0.217 | 2.254 ±0.262 | - | - |
| 30 | C25 |  | 17.582 ±0.511 | 10.116 ±0.695 | 14.190 ±0.436 | 13.168 ±0.390 |
| 31 | 13-; 11-; 9-MeC25 | 2533 | - | - | - | 0.586 ±0.053 |
| 32 | 7-MeC25 | 2541 | - | - | - | 0.198 ±0.016 |
| 33 | 5-MeC25 | 2550 | - | - | - | 0.349 ±0.024 |
| 34 | C26diene | 2559 | 0.060 ±0.014 | - | - | - |
| 35 | 9-C26ene | 2572 | - | - | 0.396 ±0.031 | 0.223 ±0.037 |
| 36 | 7-C26ene | 2582 | 0.445 ±0.039 | 0.175 ±0.048 | - | - |
| 37 | 5-C26ene | 2593 | 0.349 ±0.047 | - | - | - |
| 38 | C26 |  | 0.957 ±0.103 | 0.308 ±0.066 | 0.694 ±0.043 | 1.126 ±0.127 |
| 39 | C27diene | 2644 | - | - | 0.045 ±0.011 | 0.012 ±0.006 |
| 40 | 9,17-C27diene | 2653 | 0.197 ±0.024 | - | 0.338 ±0.044 | - |
| 41 | 7,19-C27diene | 2660 | 0.461 ±0.055 | - | - | - |
| 42 | 11-C27ene | 2672 | 0.071 ±0.021 | - | 1.265 ±0.234 | - |
| 43 | 9-C27ene | 2674 | 0.853 ±0.189 | 0.278 ±0.026 | 11.051 ±0.418 | 4.705 ±0.119 |
| 44 | 7-C27ene | 2683 | 6.721 ±0.339 | 0.632 ±0.045 | 0.805 ±0.199 | 0.830 ±0.070 |
| 45 | 5-C27ene | 2693 | 3.941 ±0.320 | 1.023 ±0.131 | - | - |
| 46 | C27 |  | 7.206 ±0.252 | 4.162 ±0.343 | 19.755 ±0.710 | 12.107 ±0.426 |
| 47 | 13-; 11-; 9-MeC27 | 2735 | - | - | - | 0.298 ±0.051 |
| 48 | 5-MeC27 | 2757 | - | - | - | 0.161 ±0.025 |
| 49 | 9-C28ene | 2774 | - | - | 0.200 ±0.025 | 0.159 ±0.027 |
| 50 | 7-C28ene | 2783 | 0.185 ±0.035 |  | - | - |
| 51 | 5-C28ene | 2793 | - | 0.0961±0.033 | - | - |
| 52 | C28 |  | 0.222 ±0.062 | 0.423 ±0.058 | 0.212 ±0.027 | 0.708 ±0.073 |
| 53 | C29diene | 2848 | - | - | 0.040 ±0.011 | - |
| 54 | C29diene_2 | 2855 | - | - | 0.018 ±0.007 | - |
| 55 | 9-C29ene | 2874 | 0.171 ±0.106 | - | 2.763 ±0.347 | 3.328 ±0.140 |
| 56 | 7-C29ene | 2884 | 0.598 ±0.071 | 0.316 ±0.036 | 0.514 ±0.167 | - |
| 57 | 5-C29ene | 2793 | - | 0.994 ±0.064 | - | - |
| 58 | C29 |  | 1.107 ±0.120 | 8.170 ±0.539 | 5.363 ±0.380 | 8.288 ±0.365 |
| 59 | 13-; 11-; 9-MeC29 | 2930 | - | - | - | 0.326 ±0.033 |
| 60 | 9-C30ene | 2974 | - | - | - | 0.128 ±0.023 |
| 61 | C30 |  | 0.144 ±0.058 | 0.449 ±0.089 | - | 0.304 ±0.037 |
| 62 | 9-C31ene | 3074 | 0.255 ±0.169 | - | 0.360 ±0.053 | 2.308 ±0.142 |
| 63 | 7-C31ene | 3083 | - | 0.383 ±0.034 | - | - |
| 64 | 5-C31ene | 3092 | - | 0.492 ±0.046 | - | - |
| 65 | C31 |  | - | 4.881 ±0.420 | 0.311 ±0.050 | 2.024 ±0.119 |
| 66 | C32 |  | 0.047 ±0.014 | 0.026 ±0.017 | - | - |
| 67 | 9-C33ene | 3273 | 0.058 ±0.025 | - | - | 0.021 ±0.007 |
| 68 | 7-C33ene | 3282 | - | 0.046 ±0.015 | - | - |
| 69 | C33 |  | - | 0.373 ±0.062 | - | - |
|  |  |  |  |  |  |  |

**Supplementary Table 3:** Pairwise PERMANOVA to compare differences in CHC profile composition between species.

| Pairs | Df | Sums of Sqs | F-Model | R² | p-value |
| --- | --- | --- | --- | --- | --- |
| *B. albopleuralis* vs *B. breviceps* | 1 | 2.240 | 97.676 | 0.699 | 0.001 |
| *B. albopleuralis* vs *B. mirus* | 1 | 2.542 | 108.37 | 0.647 | 0.001 |
| *B. albopleuralis* vs *B. prshewalskyi* | 1 | 4.893 | 239.35 | 0.794 | 0.001 |
| *B. breviceps* vs *B. mirus* | 1 | 2.155 | 128.43 | 0.767 | 0.001 |
| *B. breviceps* vs *B. prshewalskyi* | 1 | 1.812 | 141.54 | 0.771 | 0.001 |
| *B. mirus* vs *B. prshewalskyi* | 1 | 2.043 | 125.71 | 0.681 | 0.001 |

**Supplementary Table 4a:** Pairwise comparison of mean weighted chain length between species using Dunn’s test (top value – Z, bottom value – p; asterisks indicate significant values after Holm correction).

|  | *B. albopleuralis* | *B. breviceps* | *B. mirus* |
| --- | --- | --- | --- |
| *B. breviceps* | 1.73  0.08 |  |  |
| *B. mirus* | -6.47  < 0.001* | -6.54  < 0.001* |  |
| *B. prshewalskyi* | -5.11  < 0.001* | -5.50  < 0.001* | 1.48  0.07 |

**Supplementary Table 4b:** Pairwise comparison of proportion of saturated CHCs between species using Dunn’s test (top value – Z, bottom value – p; asterisks indicate significant values after Holm correction).

|  | *B. albopleuralis* | *B. breviceps* | *B. mirus* |
| --- | --- | --- | --- |
| *B. breviceps* | -1.28  0.20 |  |  |
| *B. mirus* | -2.49  0.03 | -0.60  0.28 |  |
| *B. prshewalskyi* | -4.71  < 0.001* | -2.19  0.06 | -2.10  0.05 |
